# Whole-genome sequencing of marine water-derived *Curvularia verruculosa* KHW-7: a pioneering study

**DOI:** 10.3389/fmicb.2024.1363879

**Published:** 2024-05-23

**Authors:** Payal Baranda, Shaikhul Islam, Ashish Modi, Harsh Mistry, Sami Al Obaid, Mohammad Javed Ansari, Virendra Kumar Yadav, Ashish Patel, Madhvi Joshi, Dipak Kumar Sahoo, Himanshu Bariya

**Affiliations:** ^1^Department of Life Sciences, Hemchandracharya North Gujarat University, Patan, India; ^2^Plant Pathology Division, Bangladesh Wheat and Maize Research Institute, Nashipur, Bangladesh; ^3^Department of Botany and Microbiology, College of Science, King Saud University, Riyadh, Saudi Arabia; ^4^Department of Botany, Hindu College Moradabad (Mahatma Jyotiba Phule Rohilkhand University Bareilly), Uttar Pradesh, India; ^5^Gujarat Biotechnology Research Centre (GBRC), Gandhinagar, India; ^6^Department of Veterinary Clinical Sciences, College of Veterinary Medicine, Iowa State University, Ames, IA, United States

**Keywords:** *Curvularia verruculosa* KHW-7, whole-genome sequencing, secondary metabolites, marine microorganism, functional genomics

## Abstract

Marine microorganisms are renowned for being a rich source of new secondary metabolites that are significant to humans. The fungi strain KHW-7 was isolated from the seawater collected from the Gulf of Khambhat, India, and identified as *Curvularia verruculosa* KHW-7. On a next-generation sequencing platform, *C. verruculosa* KHW-7’s whole-genome sequencing (WGS) and gene annotation were carried out using several bioinformatic methods. The 31.59 MB genome size, 52.3% GC, and 158 bp mean read length were discovered using WGS. This genome also contained 9,745 protein-coding genes, including 852 secreted proteins and 2048 transmembrane proteins. The antiSMASH algorithm used to analyze genomes found 25 secondary metabolite biosynthetic gene clusters (BGCs) that are abundant in terpene, non-ribosomal peptide synthetase (NRPS), and polyketides type 1 (T1PKS). To our knowledge, this is the first whole-genome sequence report of *C. verruculosa*. The WGS analysis of *C. verruculosa* KHW-7 indicated that this marine-derived fungus could be an efficient generator of bioactive secondary metabolites and an important industrial enzyme, both of which demand further investigation and development.

## Introduction

*Curvularia* is a genus of fungi that includes several species, many of which are plant pathogens. This fungal genus is recognized for causing diseases in several economically important agricultural crops, including maize, wheat, barley, rice, and grasses (Huang et al., 2005; Shirsath et al., 2018; Wang et al., 2022). Moreover, this fungus can also cause various types of infections in humans and animals (Samaddar et al., 2023). *Curvularia* infections can range from moderate to severe and affect numerous regions of the body, including the skin, lungs, and nails (da Cunha et al., 2013). To date, there are 131 species of *Curvularia* reported worldwide as per the list of Fungorum (Quach et al., 2022). Among these species, *C. verruculosa* is a significant plant pathogen associated with causing various diseases such as leaf spot, blight, and ear rot in different plant species (Wei et al., 2022). These diseases can lead to significant yield losses in agricultural production. It has a wide geographical distribution, impacting crops in diverse regions globally, particularly in warm and humid climates where conditions favor its growth and spread. The fungi can survive in soil for a long time and infect plants through their roots or wounds on their stems or leaves. Symptoms of *C. verruculosa* infection vary among crops but commonly include leaf lesions, discoloration, wilting, and, in severe cases, the rotting of seeds or ears (Rajput et al., 2020). Managing diseases caused by *C. verruculosa* can be challenging. The pathogen exhibits certain resistance to fungicides, and its control often relies on integrated management practices involving cultural, biological, and chemical measures.

Given its ability to affect staple food crops, *C. verruculosa* can pose a threat to food security, especially in regions highly reliant on these crops for sustenance. Understanding the significance of *C. verruculosa* as a plant pathogen is crucial for implementing appropriate disease management strategies and developing resistant crop varieties to mitigate its impact on agricultural productivity. As a result, traditional techniques are insufficient to determine the boundaries of a species within their respective genus, and complete genome sequencing is required for proper identification and characterization.

Whole-genome sequencing (WGS) involves decoding an organism’s full DNA sequence, providing a comprehensive grasp of its genetic backbone. In fungi, WGS plays a crucial role in their characterization and offers several significant advantages (Tao et al., 2022). WGS allows for the assessment of the entire genetic diversity within a fungal species. It helps identify variations in genes responsible for traits such as virulence, pathogenicity, and fungicide resistance. For fungi, accurate taxonomic classification is essential. WGS enables precise species identification and phylogenetic analysis, contributing to a better understanding of fungal evolution and relationships between different species (Quach et al., 2022). By analyzing the entire genome, it is possible to pinpoint specific genes or gene clusters associated with virulence and pathogenicity in fungi. WGS helps in detecting genetic markers associated with antifungal resistance. This information is critical in guiding treatment strategies and developing new antifungal substances to combat resistant strains. By comparing multiple fungal genomes, scientists can identify conserved regions and unique genes, shedding light on species-specific characteristics and potential targets for diagnostics or therapeutics. WGS aids in tracking outbreaks, understanding transmission patterns, and differentiating between strains or isolates. However, only seven genomes of other *Curvularia* species are accessible through the GenBank (NCBI) database. The genome of the plant pathogen *C. verruculosa* was previously sequenced to facilitate in-depth evolutionary research and enhance our understanding of pathogen origin and infection processes. The results of this study will contribute to the existing *Curvularia* genome database and facilitate future investigations into its pathogenic nature.

## Materials and methods

### Sample collection, isolation, and identification of fungi

The marine water sample was collected from four divergent spots in the sea area of the Gulf of Khambhat, India (22.1775N 72.4763 E) (Supplementary Figure S1). Sea water was collected 10 m apart from each spot and at 1 m depth in a germ-free container and transported to the laboratory under cool conditions for further isolation and purification of fungi. Fungi from collected samples were isolated using a marine agar medium (Bonugli-Santos et al., 2015) by adopting serial dilution followed by incubation for 48–96 h at 25°C. Single and pure fungal colonies were picked up and further allowed for growth on marine agar plates. A pure fungal strain, KHW-7, was identified by morphological and microscopic observation as well as by sequencing of amplified ITS region of the fungal gene.

### DNA isolation and quality analysis

**Figure 1:**
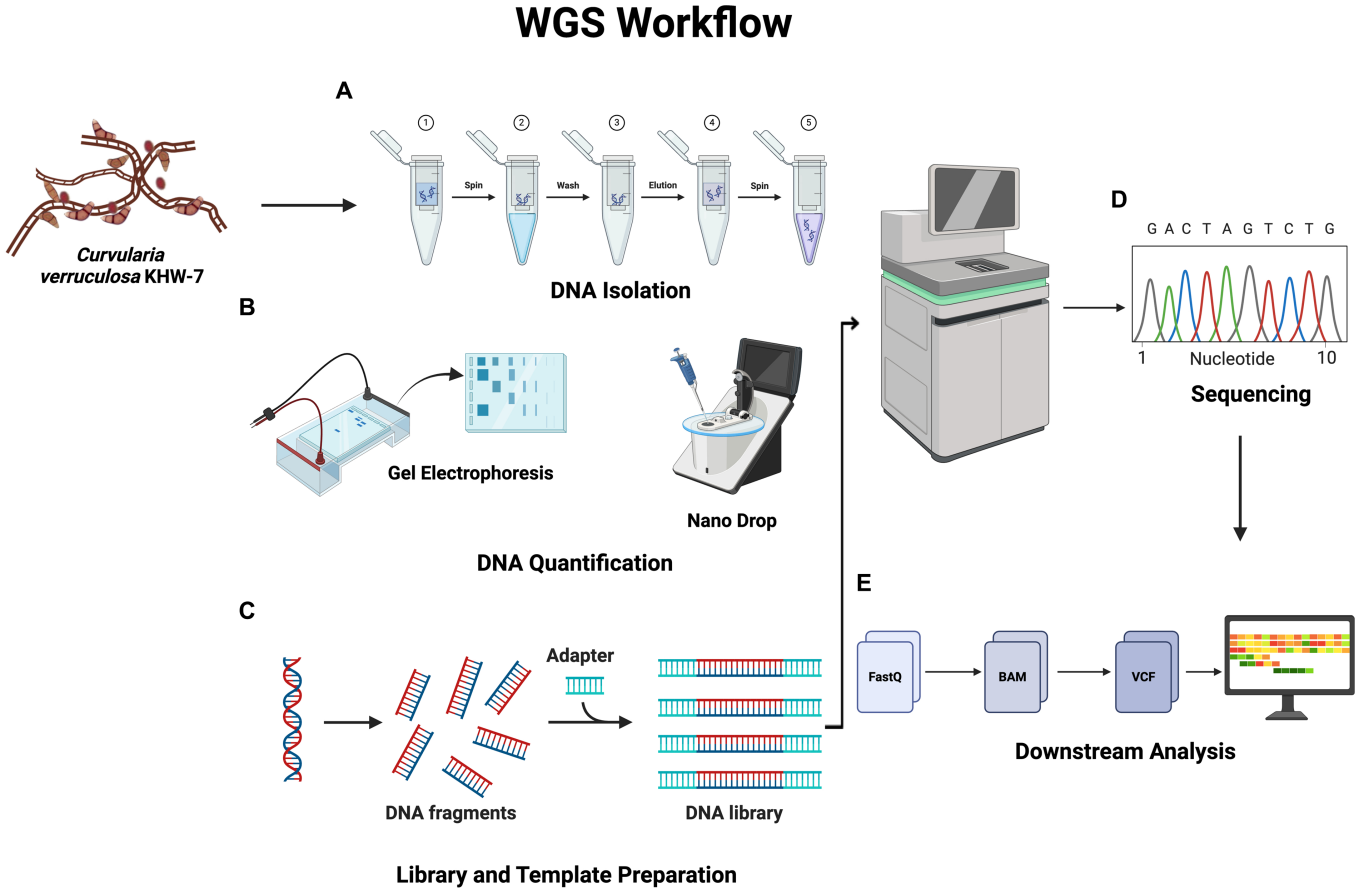
Workflow showing whole-genome sequencing. DNA quantification is carried out following the samples’ DNA isolation. The latter stages of the WGS procedure are sequencing and library preparation. Finally, several bioinformatic techniques are used to carry out downstream analysis. The figure was generated using BioRender (www.biorender.com; accessed on 23rd April 2024).

The workflow for the WGS experiments is shown in Figure 1. Genomic DNA from the KHW-7 strain was extracted using the silica spin column DNA extraction method following the manufacturer’s manual. Subsequently, 08% agarose gel electrophoresis was performed to check the quality of isolated genomic DNA. The presence of a single intact band within the gel matrix is indicative of the superior quality of isolated genomic DNA. Additionally, a 2-μl fraction of the genomic DNA sample was subjected to spectrophotometric analysis using the BioTeK Epoch spectrophotometer to determine the A260/280 ratio. This ratio serves as a pivotal measure of DNA purity.

### Library preparation and sequencing

The libraries were prepared using the commercially available Ion Xpress^™^ Plus Fragment Library Kit (Thermo Fisher Scientific, United States) as per the manufacturer’s instructions. This process involved stages such as DNA fragmentation, fragment purification, ligation of fragments, fragment amplification, and final quantification. The commercially available Ion Library TaqMan Quantitation kit was used for quantification purposes. The size of the fragmented DNA was assessed (Quality Control Step) using the Agilent^™^ High Sensitivity DNA Kit on the Agilent^™^ 2,100 Bioanalyzer, following the provided instructions. After library preparation, the template was prepared using the Ion Chef automated system according to the manufacturer’s instructions available with the Ion 550 Kit (Thermo Scientific, United States), and the Ion 550 Chip Kit was utilized for loading samples with the assistance of the Ion Chef, followed by sequencing on the Ion GeneStudio S5 Plus System (Ion Torrent, Thermo Scientific, United States).

### Preannotation data processing

The Ion Torrent single-end sequencing reads were subjected to adapter and quality trimming using cutadapt (v4.7) and Trim Galore (v0.4.1) with phred score cutoff of 20. The obtained superior quality reads were built from scratch using SPAdes v3.13.0. The initial analysis, such as base pair calling and trimming of sequences, was performed using the Ion Torrent browser. This process resulted in obtaining readings of good quality. The sequence readings were assembled *de novo* using the SPAdes assembler v3.1.0 (Torrent browser) with the default parameters. The scaffolds obtained were filtered based on their respective length, with a minimum threshold of 500 base pairs. Assembly statistics were generated by QUAST (v5.2.0).

### Gene annotation

A repeat library was created from scratch for the chosen assembly of the KHW-7 strain using RepeatModeler v2.0.4. This library was then utilized as a customized library for softmasking with RepeatMasker v4.1.5. The Funannotate v1.8.16 pipeline was used to structurally annotate the masked assemblies. The BUSCO database v5.7.1 was utilized to identify conserved gene models for the purpose of training the *Ab initio* gene predictors such as Augustus, glimmerhmm, and snap. The generation of gene models was based on evidence, achieved by matching the sequences of contigs with the unified protein sequence database (UniProtKB; https://www.uniprot.org/) using the DIAMOND program. Subsequently, the gene models were refined using Exonerate. The Funannotate process utilized the EVidenceModeler,^1^ which included a weighting method, to choose the consensus models from a pool of *ab initio* and evidence-based gene models. Functional annotation of the consensus models was conducted following the elimination of models with insufficient lengths, gaps, and transposable elements (TEs).

The gene models were functionally predicted using InterProScan (v-5.67-99.0), which involved mapping to the Gene Ontology (GO) database^2^ and eggNOG-mapper (v4.5.1) based on the eggNOGorthology database.^3^ The significantly enriched GO terms were further analyzed to find out the interactions among several biosynthetic pathways using the “ClueGO”^4^ plugin of the CytoScape software (v3.7.2.0). Signal peptides (secretome) were predicted using SignalP (v6.0) and Phobius.^5^ Biosynthetic gene clusters (BGCs) were identified in the genome using fungiSMASH,^6^ which is a specialized version of antiSMASH designed for fungal genomes. The tRNA and rRNA genes were detected using tRNAscan-SE^7^ and Barrnap,^8^ respectively.

Functional annotation using the annotate pipeline^9^ used to annotate genes by performing similarity search against databases of UniProt, Pfam, dbCAN (CAZyme), MEROPS, and BUSCO pezizomycotina gene models. Output of InterPro, EggNog, SignalP, Phobius, tRNAscan-SE, and antiSMASH was added to the final comprehensive annotation files, which can be directly submitted to the National Center for Biotechnology Information (NCBI). Further annotation was conducted using the NCBI non-redundant (NR) genome database,^10^ Pathogen Host Interactions (PHIs),^11^ and the Comprehensive Antibiotic Resistance Database (RGI-CARD).^12^

## Results

### Fungal strain identification

The fungal isolate, KHW-7, was identified using ITS gene sequencing. This partial ITS gene sequence showed 100% homology with reference strains of *C. verruculosa*. Barrnap was used to predict rRNA genes within the assembled genome. Partial ITS gene sequence was searched in the predicted 9 rRNA sequences of KHW-7 using local NCBI blastn application, and Blast output produced 100% alignment against contig574 spanning 5.8S region as well as between start and end of 18S and 28S rRNA regions, respectively. Other phylogenetic marker genes, glyceraldehyde 3-phosphate dehydrogenase (gdph), found in the annotated protein sequences, show 100% similarity with other partial gdph genes of *C. verruculosa*.

The taxonomy of this genome is:

Cellular organisms >Eukaryota >Opisthokonta >Fungi >Dikarya >Ascomycota >saccharomyceta >Pezizomycotina >leotiomyceta >dothideomyceta >Dothideomycetes >Pleosporomycetidae >Pleosporales > Pleosporineae >Pleosporaceae > Curvularia >*Curvularia verruculosa.*

### Sequencing and assembly of the genome

**Figure 2:**
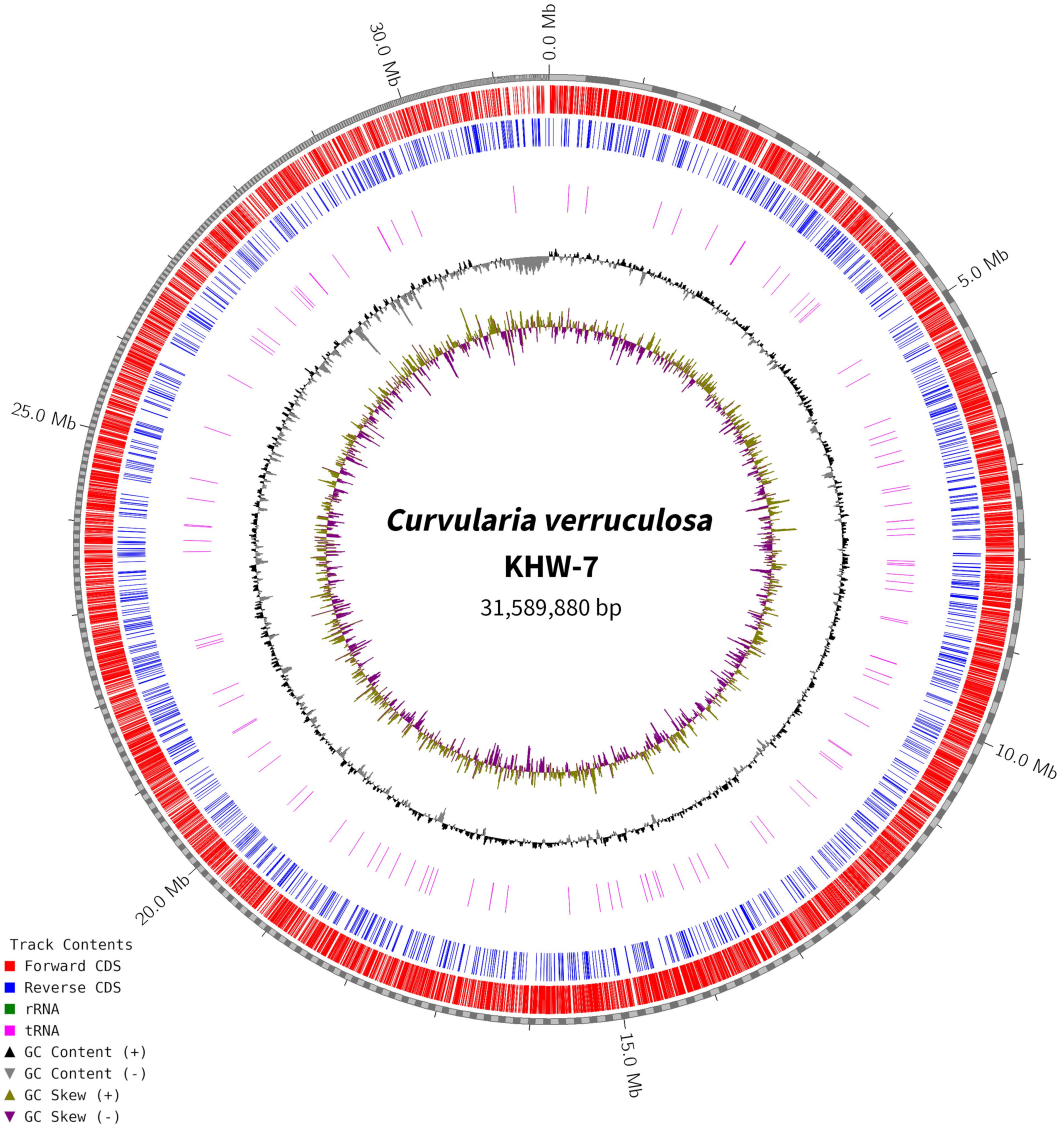
Circos plot of the annotated genome of *C. verruculosa* KHW-7. The forward and reverse CDS. rRNA, tRNA, and GC contents are shown.

For genome assembly, the SPAdes assembler, version 3.13.0, was used to process and utilize a total of 6,110,868 high-quality reads. The 31.59 Mb genome of *C. verruculosa* KHW-7 featured a guanine–cytosine (GC) content of 50.44%. The screened reads were ordered into 1,323 contigs (≥500 bp), making 31,589,880 bp large genome with 81,050 N50 value. Moreover, the integrity was 97%, indicating excellent quality of genome assembly. The circos plot of the annotated genome of *C. verruculosa* KHW-7 is depicted in Figure 2. Another reported *Curvularia* spp. was also reported to have a genome size of 33–36 Mb and an average 50% G + C content (Quach et al., 2022). Comparative genome features of reported *Curvularia* spp. are depicted in Table 1. At the species level, *C. verruculosa* KHW-7 is the first sequenced whole genome. The presence of repetitive elements, such as interspersed repeats and low-complexity DNA sequences, in the genome assembly is significant due to their recognized involvement in genome length expansion and evolution (Sun et al., 2012). Annotation of the draft genome using funannotate pipeline and other RNA prediction tools predicted a total of 9,877 genes, of which 9,745 mRNA (CDSs), 123 tRNA, and 9 rRNA were predicted. As per the funannotate pipeline, complete CDSs were 9,489, and partial CDSs were 256. Predicted multiple exon transcripts were 7,541 and single exon transcripts were 2,204, with an average protein length of 502.68. TEs are mobile genetic elements that have a role in the occurrence of mutations, regulation of gene expression, and rearrangement of chromosomes, enabling populations to adapt efficiently to environmental changes (Lorrain et al., 2021). A total of 9,877 genes were functionally annotated by performing sequence similarity searches against the Pfam, InterPro, BUSCO, EggNOG, MEROPS, and CAZyme databases and utilizing the SignalPsecretome prediction program. These searches resulted in a total of 25,199 annotations. Further, the KEGG annotation predicts a total of 3,724 genes, the COG annotation predicts a total of 6,979 genes, and the GO terms annotation predicts a total of 6,318 genes. (Table 2). Supplementary Table S1 shows the comparison of genome features between *C. verruculosa* KHW-7 and other *Curvularia* species.

**Table 1:** General features of the *C. verruculosa* KHW-7 genome.

Features	Number/bp
Total raw bases	1,023,799,652 bp
Good quality (>Q20) bases	849,468,143 bp
Total raw reads (sequences)	6,276,793
Good quality reads (sequences)	6,133,114
Number of assembled reads (sequences)	6,110,868
Mean read length	158 bp
Mean GC percent	52.3%
Contigs (≥ 500 bp)	1,323
Total length	31,589,880 (31.59 Mb)
Largest contig	390,585 bp
GC (%)	50.44
N50	81,050
N90	18,713
L50	120
L90	409

**Table 2:** Complete gene prediction and functional annotation of *C. verruculosa* KHW-7 genome.

Gene prediction and annotation parameters		Number
Gene prediction	Total nos. of genes	9,877
	Mean gene length (bp)	1638.3
	Genome % covered by genes	46.61
	Total nos. of proteins	9,745
	tRNA	123
	rRna	9
	Complete CDSs	9,489
	Partial CDSs	256
Secretome prediction	Secreted proteins	852
	Transmembrane proteins	2048
Secondary metabolites	T1PKS, NRPS, Terpene, etc.	25
Functional annotation	Pfam	6,964
	CAZyme	509
	Merops	326
	Busco	1,262
	Gene ontology	6,318
	InterProScan	7,316
	Eggnog	7,970
	NCBI NR (protein database)	9,651

### EggNOG annotation

**Figure 3:**
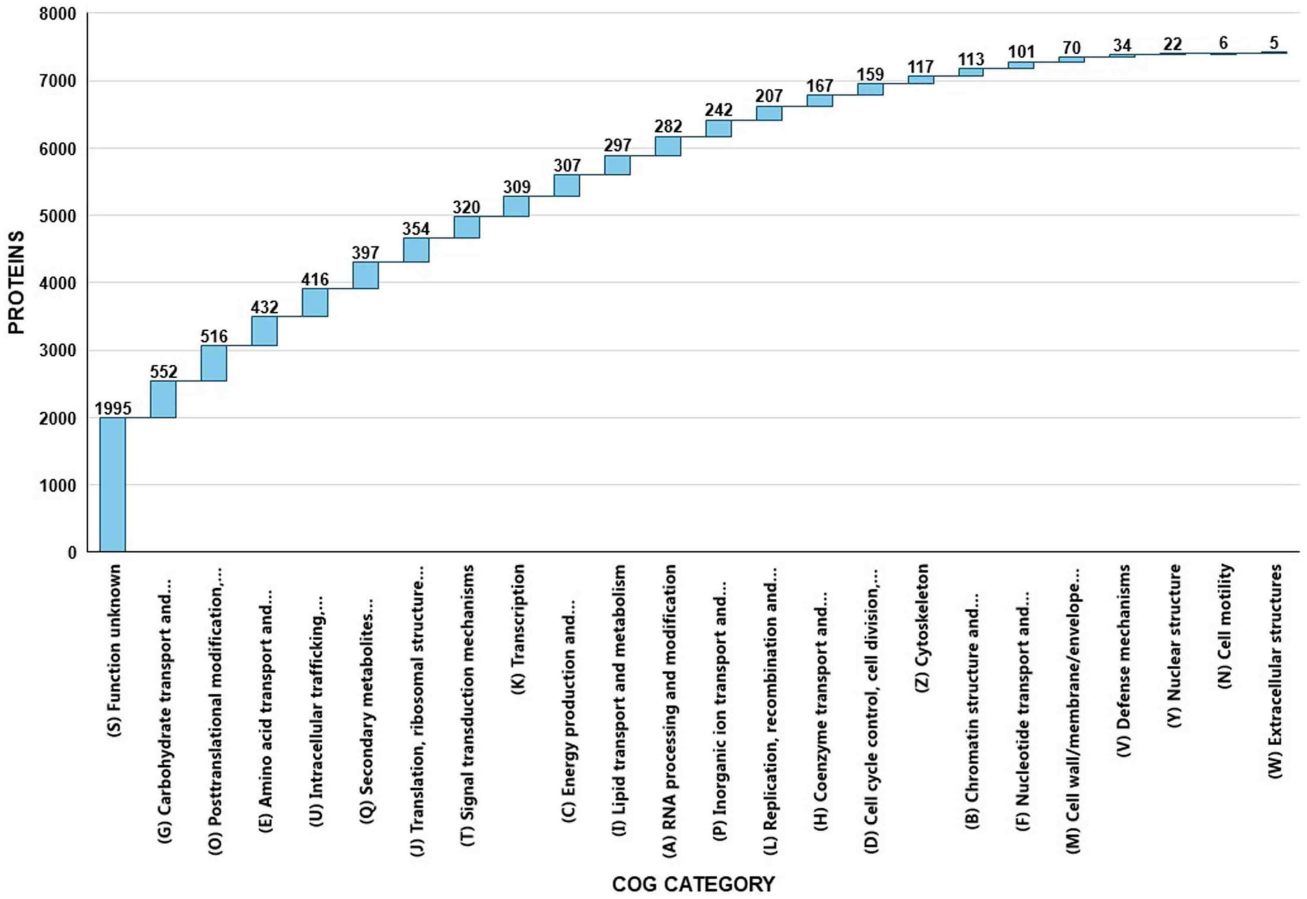
EggNOG COG annotation of *C. verruculosa* KHW-7. Proteins related to carbohydrate transport, posttranslational modifications, amino acid transport, and intracellular trafficking were significantly enriched.

**Figure 4:**
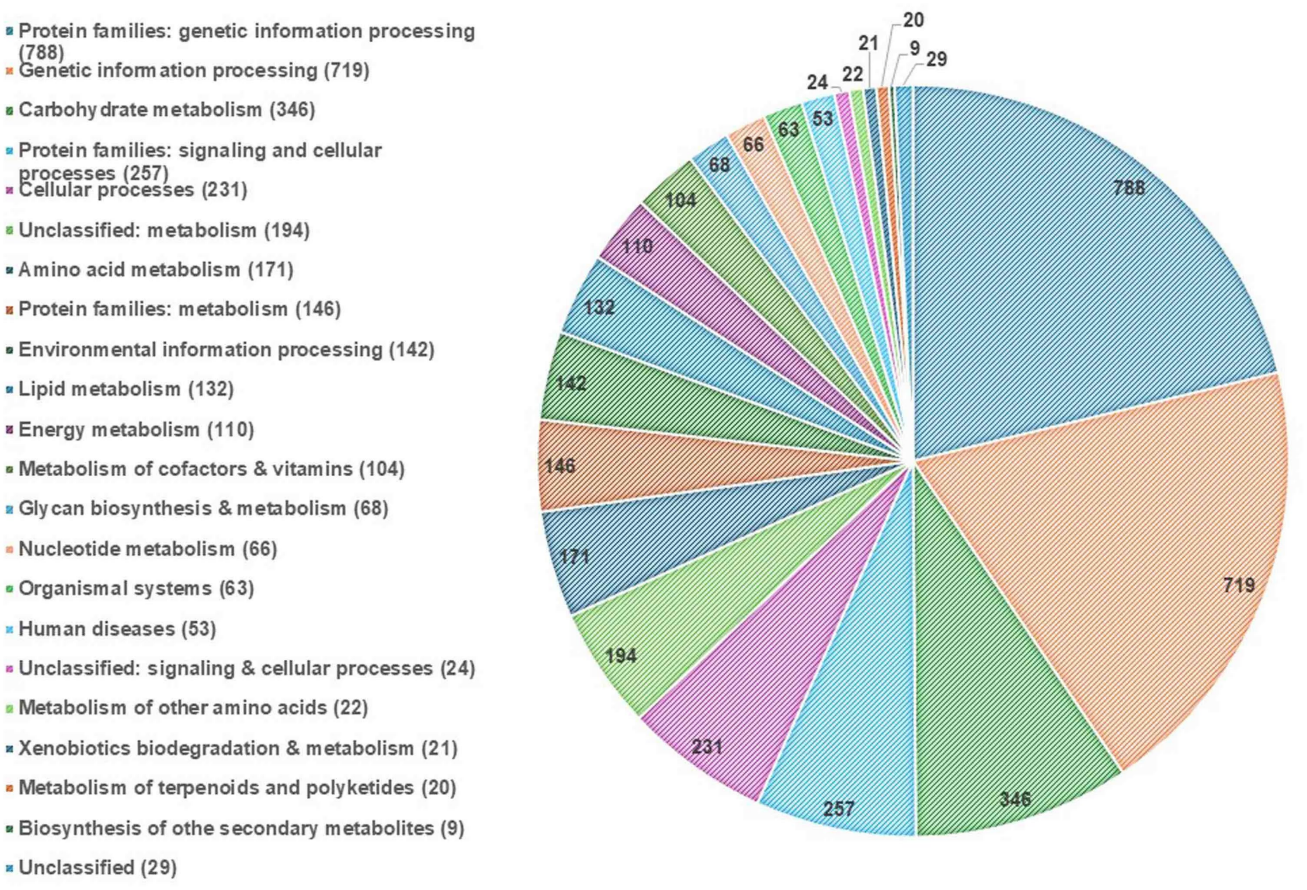
KEGG pathway annotation. The highest enrichment was found in the protein families related to genetic information processing, followed by carbohydrate metabolism.

EggNOG-mapper annotated 75% of the predicted proteins in the genome, assigning them to 6,979 eggNOGorthogroups, which represent over 24 functional categories. The most abundant functional categories were S, G, O, U, and E. These results suggest that *C. verruculosa* KHW-7 is well-adapted to carbohydrate metabolism, protein turnover, and intracellular trafficking. The annotation comprised 991 putative proteins and 6,979 proteins with confirmed functions. The proteins were categorized as follows: 1,771 proteins were assigned Enzyme Commission (EC) numbers, 3,374 proteins were assigned GO assignments, and 3,803 proteins were linked to the KEGG pathways. EggNOG annotated 6,964 proteins using Pfam, a curated protein domain family (Figure 3). While EggNOG provides a reliable and precise genome annotation, the approach and execution of the annotation differ conceptually from that of BlastKoala (KEGG). Thus, the functional annotation was conducted utilizing the anticipated protein sequences of the genome. BlastKoala annotated 3,724 entries (38.2%) from a total of 9,868 entries (protein sequences), and 403 pathways were classified into 22 functional categories as per Figure 4 and Table 3.

**Table 3:** KEGG pathway prediction.

Functional categories	Entries
Protein families: genetic information processing	788
Genetic information processing	719
Carbohydrate metabolism	346
Protein families: signaling and cellular processes	257
Cellular processes	231
Unclassified: metabolism	194
Amino acid metabolism	171
Protein families: metabolism	146
Environmental information processing	142
Lipid metabolism	132
Energy metabolism	110
Metabolism of cofactors and vitamins	104
Glycan biosynthesis and metabolism	68
Nucleotide metabolism	66
Organismal systems	63
Human diseases	53
Unclassified: signaling and cellular processes	24
Metabolism of other amino acids	22
Xenobiotics biodegradation and metabolism	21
Metabolism of terpenoids and polyketides	20
Biosynthesis of the secondary metabolites	9
Unclassified	29

### Interproscan and go annotation

**Figure 5:**
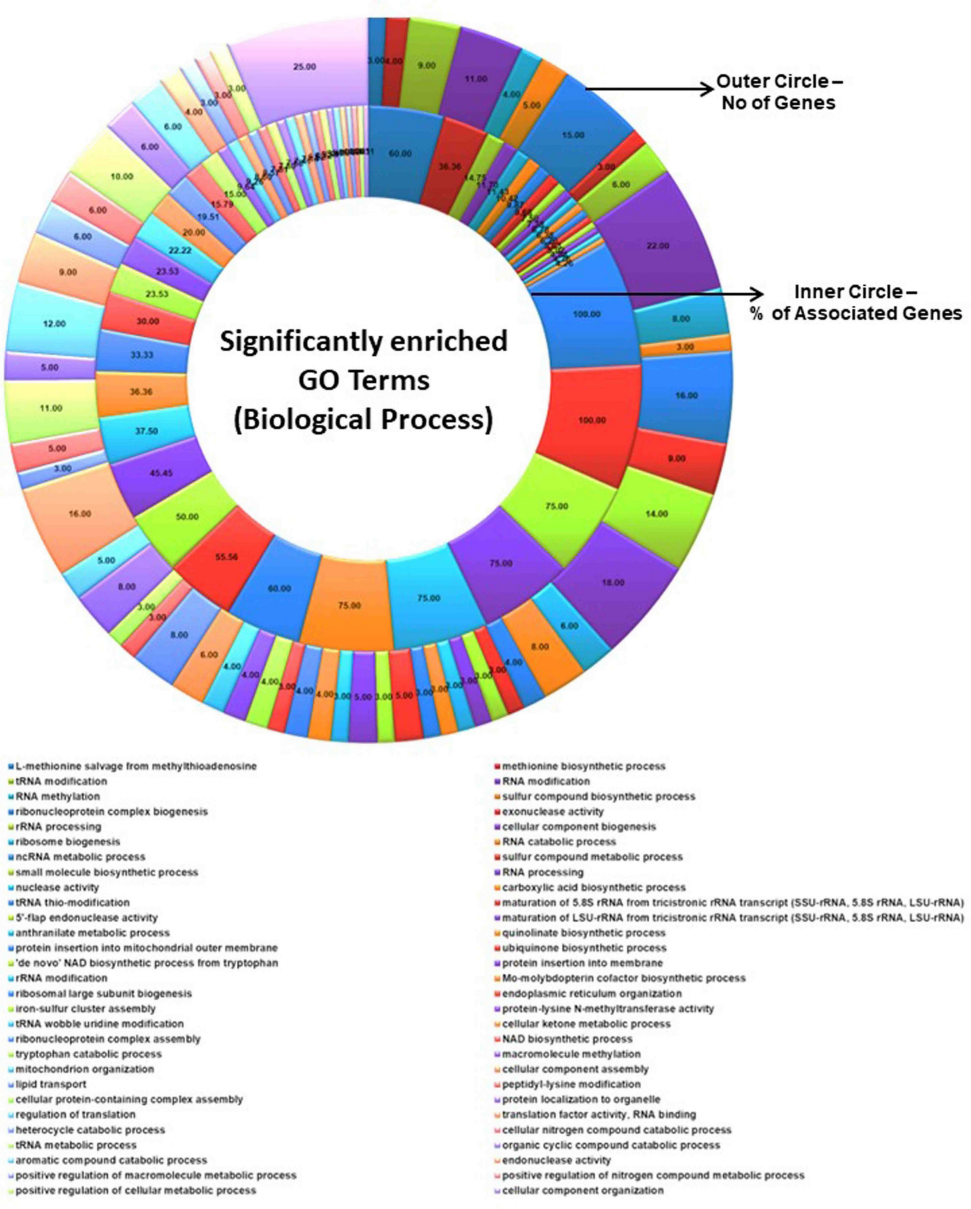
Significantly enriched GO terms (biological process). The total number of genes associated with a specific GO term and % of associated genes are shown.

**Figure 6:**
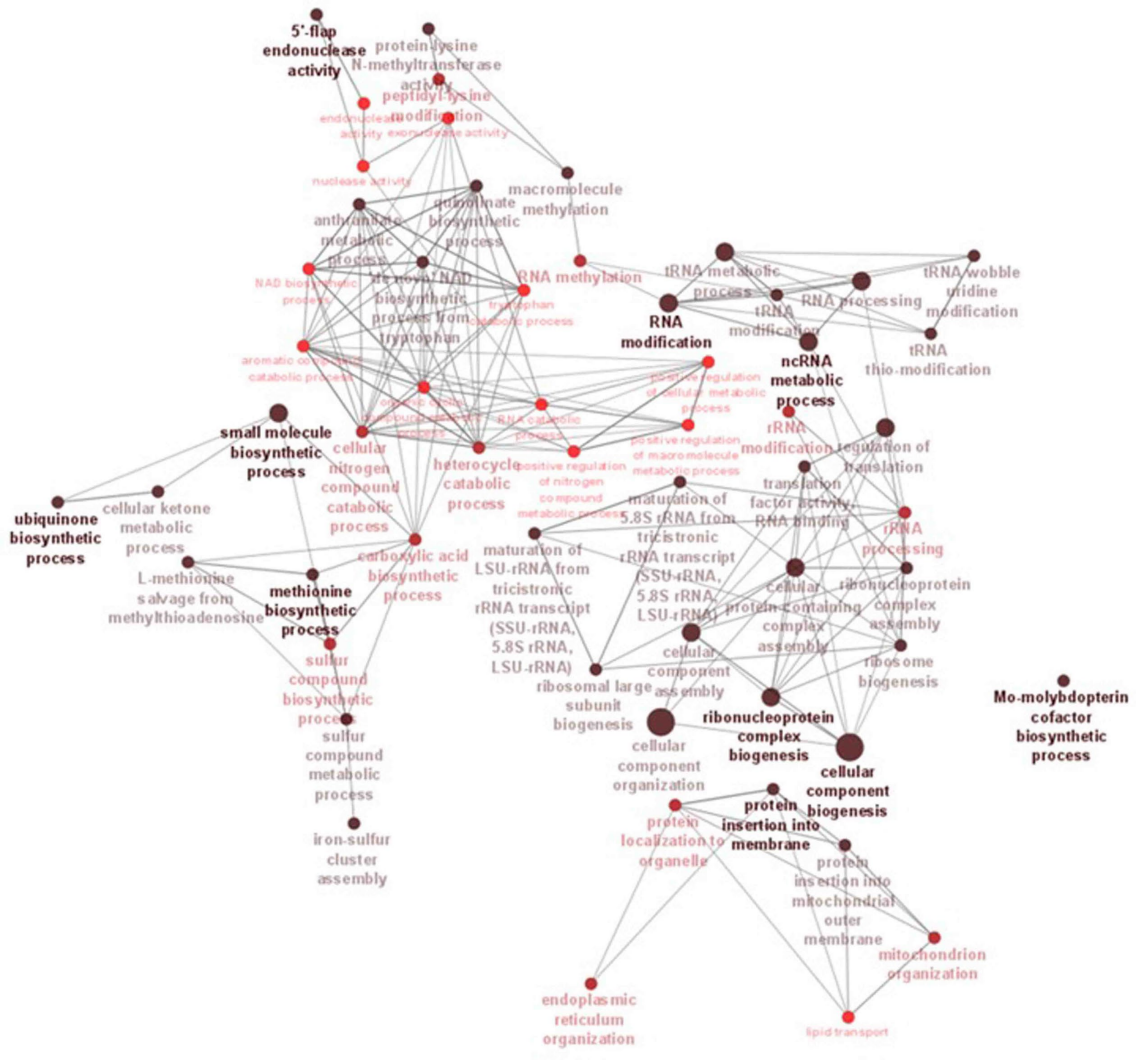
ClueGO network analysis results of the significantly enriched GO terms (biological process). This analysis demonstrates the strong connections between biological processes that could have a substantial impact on the biology of *C. verruculosa* KHW-7. These activities include the biosynthesis of methionine and ubiquinone, 5′-flap endonuclease activity, RNA alterations, and protein insertion into membranes.

InterProScan consolidates protein signatures from many databases into a unified and searchable resource, leveraging their unique capabilities to create a robust integrated database and diagnostic tool for classifying protein sequences. InterProScan classifies proteins into families and identifies important domains and sites, which is invaluable for identifying distantly related proteins and predicting their functions. Interproscan has annotated a total of 9,489 genes out of a total of 9,745 genes predicted, of which 6,318 are with GO annotation. A total of 3,056 genes were assigned the Enzyme Commission (EC) codes (Supplementary Figure S2; Table 4). A more precise identification of the interaction among different biosynthetic pathways was performed by the CytoScape network analysis of the various significantly enriched GO terms (biological process, molecular function, and cellular component), as shown in Figures 5, 6 and Supplementary Figures S3, S4.

**Table 4:** GO category summary of *C. verruculosa* KHW-7.

GO Classification	GO counts	Associated genes
Biological process	15,364	4,758
Molecular function	9,319	5,372
Cellular component	10,439	4,282

### Secondary metabolites

The antiSMASH, specifically the fungal version (fungiSMASH), analysis of *C. verruculosa* genome revealed 25 BGCs for secondary metabolites, of which 9 regions show sequence similarity from 13 to 100%. *C. verruculosa* is found to be rich in T1PKS (Polyketides type 1) (6 hits, 13 to 100%) and more so than with other secondary metabolites’ signals, such as non-ribosomal peptide synthetase (NRPS) (2 hits, 46 and 50%) and terpene (40%) (Supplementary Figure S5). The gene located at position 73.1 exhibited a significant similarity with the choline biosynthesis gene cluster (GenBank: CH236925.1) from *Aspergillus nidulans* FGSC A4. Prior reports indicate that administering choline and alpha-lipoic acid to Balb/c mice resulted in a significant reduction in the levels of isoprostanes and reactive oxygen species (ROS) produced in bronchoalveolar lavage (BAL) fluid. This, in turn, effectively regulated oxidative stress. The administration of either choline or alpha-lipoic acid resulted in a decrease in lipid peroxidation levels and NFkappaB activity, as demonstrated by Mehta et al. (2009). Therefore, these compounds can be regarded as major antioxidants. In fungi, metabolites play a crucial role in the proliferation of filamentous fungi (Markham et al., 1993). The genome BGC region 88.1 of *C. verruculosa* exhibited significant resemblances to the *Glarea lozoyensis* 1,3,6,8-tetrahydroxynaphthalene BGC (GenBank: AF549411.1). The analysis suggests that the T1PKS gene cluster present in the genome of *C. verruculosa* could be accountable for the synthesis of 1,3,6,8-tetrahydroxynaphthalene (T4HN). The study conducted by Mosunova et al. (2022) showed that melanin-forming fungus actively synthesizes T4HN using the acetogenic pathway. T4HN was identified as a result of the pentaketide synthase PKS1 in the black fungus *Colletotrichum lagenarium*. Several instances of BGCs have been documented in *Aspergillus* section *Nigri*, which is mostly linked to the production of bioactive secondary metabolites (Wang et al., 2023). Region 393.1 was discovered to bear a strong resemblance to the peramine BGC observed in *Epichloe festucae* (GenBank: AB205145.1). Epichloë synthesizes peramine, a compound that exhibits antibacterial, fungicidal, and insecticidal properties. This measure protects crops post-harvest by effectively countering phytopathogenic organisms (Song et al., 2021).

### Carbohydrate enzymes (CAZyme)

**Figure 7:**
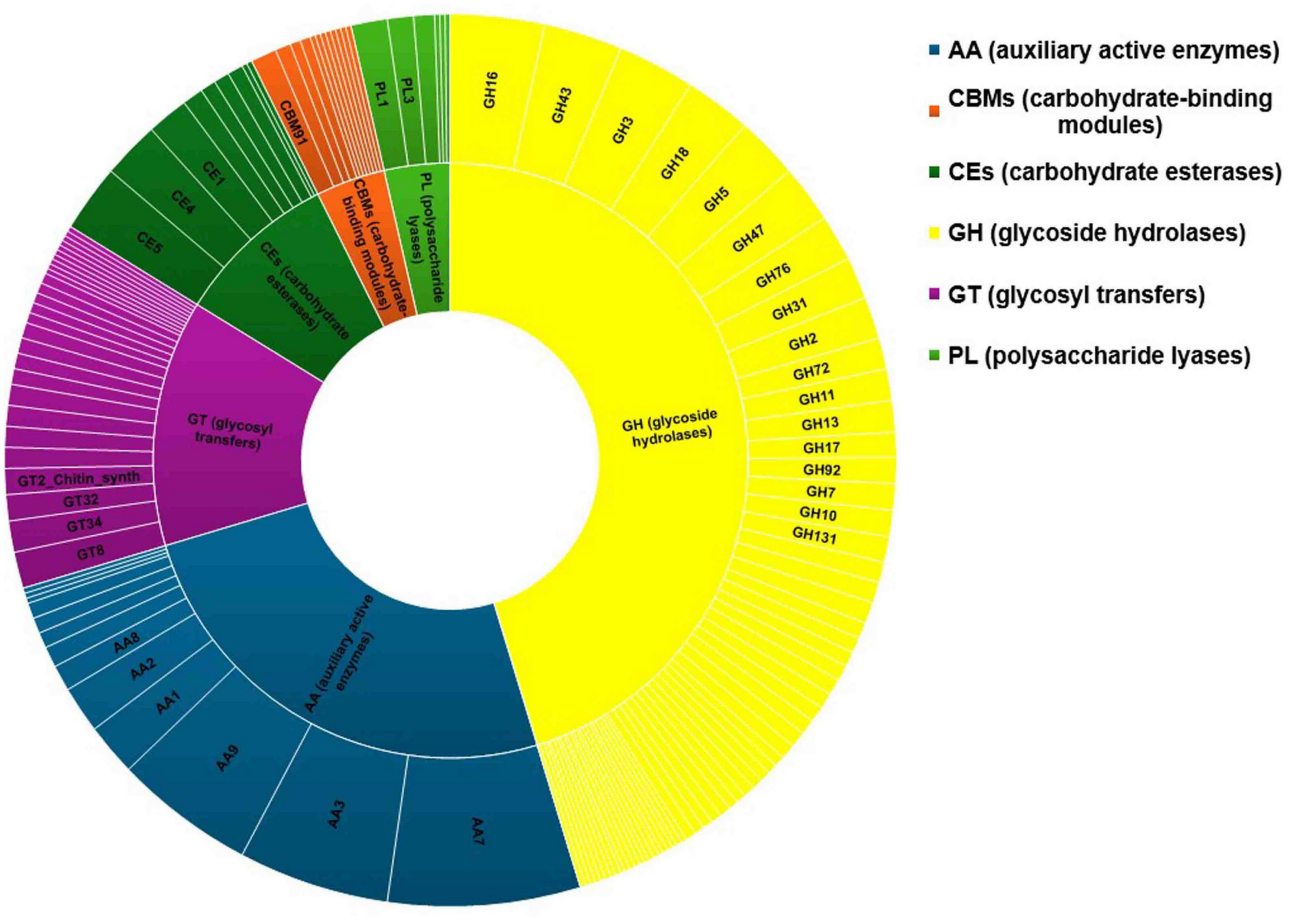
Carbohydrate enzymes identified from the *C. verruculosa* genome. Most of the carbohydrates belonged to glycoside hydrolases followed by auxiliary active enzymes.

The fungal genome possesses CAZymes gene families, which are widely responsible for many biological events, including the degradation of lignocellulose materials (Garron and Henrissat, 2019). Functional annotation of the genes of *C. verruculosa* was conducted using the CAZy database. CAZy is a specialized database for data annotation that focuses on carbohydrate enzymes (Brandi et al., 2009). There were 509 genes identified as CAZymes, and they were classified into six different kinds in the database. The genes were ranked in descending order based on their abundance, with glycoside hydrolases (GHs) having the highest count of 240, followed by auxiliary activities (AAs) with 133, glycosyl transfers (GTs) with 71, carbohydrate esterases (CEs) with 47, carbohydrate-binding modules (CBMs) with 20, and polysaccharide lyases (PLs) with 19 (Figure 7). Moreover, 47.15% of total genes of CAZymes were occupied by GHs, followed by auxiliary active enzymes (AAs) (26.12%), establishing these fungias potent strain for the breakdown of biomaterials mainly composed of lignin, cellulose, and hemicelluloses (Chylenski et al., 2019). The presence of CEs, CBMs, and PLs also confirms various plant biomass degradation capacities of *C. verruculosa* (Geng et al., 2021). The comparative analysis of CAZymes from all studied genomes of *Curvularia* spp. showed similar patterns of CAZymes found in *C. verruculosa* KHW-7 (Quach et al., 2022). Other than GHs and AAs, the genome of *C. verruculosa* KHW-7 also possesses 71 GTs family members largely involved in the biosynthesis of various polysaccharides and oligosaccharides (Lairson et al., 2008).

### Peptidase database and transcription factors

**Figure 8:**
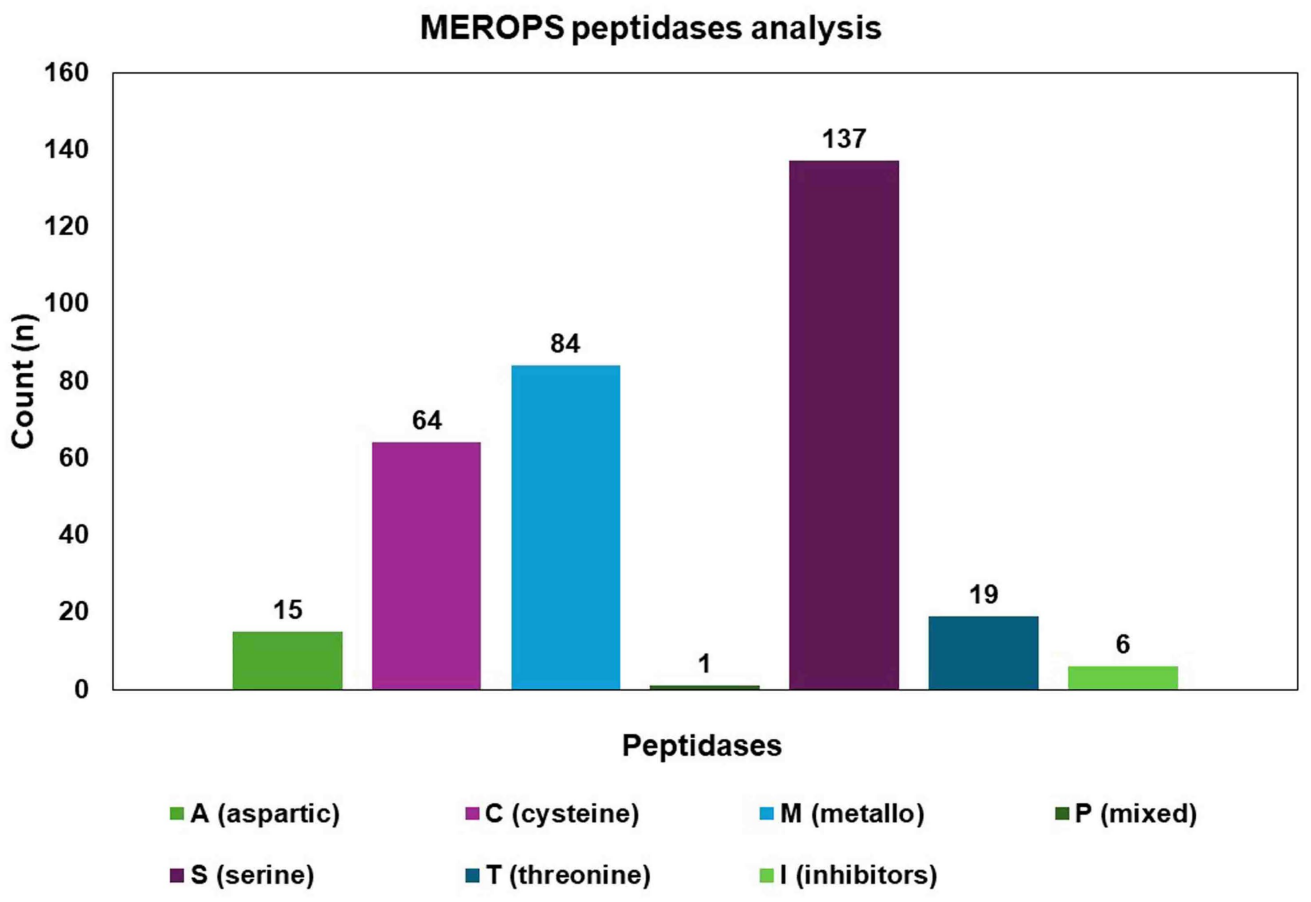
Identified proteases from the *C. verruculosa* genome. Cysteine was found to be the most enriched protease, followed by metalloprotease.

**Figure 9:**
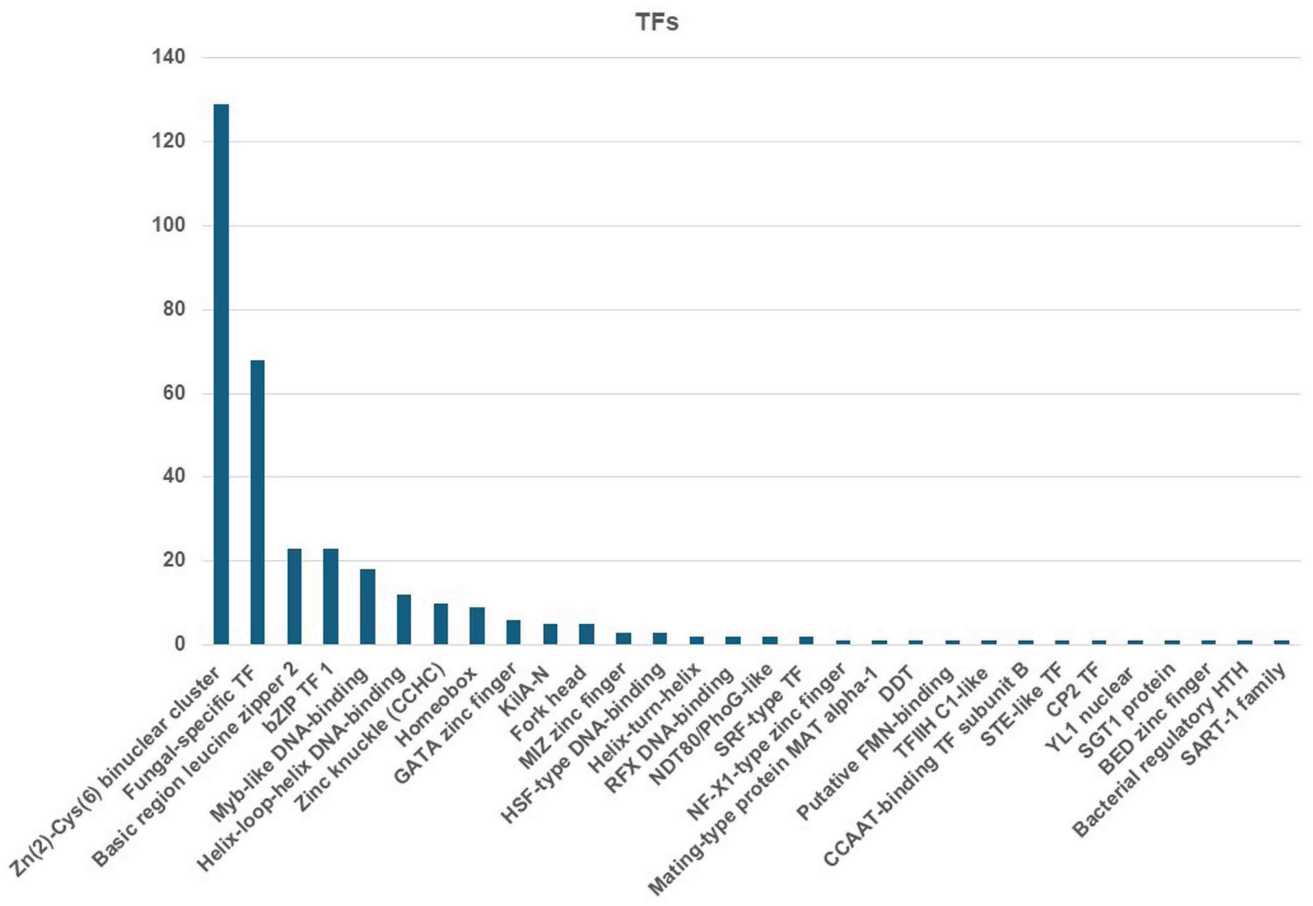
Identified TFs from the *C. verruculosa* genome. The fungal Zn(2)-Cys(6) binuclear cluster domain (IPR001138) was the most abundantly present TFs.

The MEROPS database discovered a total of 326 proteases, which may be classified into different groups, including aspartic (A), cysteine (C), metallo (M), serine (S), mixed (P), and threonine (T). Additionally, the database also includes a class for protease inhibitors (I), as depicted in Figure 8. The two highest ranking families among the transcription factor (TF) families were the fungal Zn(2)-Cys(6) binuclear cluster domain (IPR001138) and the fungal-specific TF domain (IPR007219) (Figure 9).

### Genes regulating pathogenicity factors

The Comprehensive Antibiotic Resistance Database (CARD) is designed as an antibiotic resistance ontology (ARO) that links antibiotic modules with their targets, resistance mechanisms, gene variants, and other relevant information. The Resistance Gene Identifier has given no hits against the CARD database. Hence, no drug resistance genes are present in the *C. verruculosa* KHW-7 genome.

Genes associated with pathogenicity factors were examined and identified utilizing the Pathogen Host Interaction (PHI) database. Sequences from the PHI database were downloaded from the Virulence Factor Database (VFDB). The total number of protein sequences in the full database was 8,216. A BLAST homology search between the DNA sequences of *C. verruculosa* strain KHW-7 and PHI database proteins revealed a total of 136 hits (Supplementary Table S2).

The analysis has revealed that critical pathogenic genes exhibit a variety of interacting behaviors, including an increase in virulence (4 genes), lethal (5 genes), unaffected pathogenicity (35 genes), and key non-pathogenic/low virulence genes, including the loss of pathogenicity (17 genes) and reduced virulence (69 genes), are detailed in Supplementary Table S2.

## Discussion

Due to advancements in next-generation sequencing technology, there has been a growing focus on studying fungal genomes because of their intricate genomic and physiological characteristics. This paper is the initial publication of a complete genome sequencing of *Curvularia verruculosa*, a widely recognized plant pathogen. The findings enhance our comprehension of the genetic characteristics of *C. verruculosa*, particularly in relation to the synthesis of diverse metabolites and their components contributing to pathogenicity. Presently, there are a mere eight *Curvularia* genomes that have been sequenced and stored in the GenBank database maintained by the National Center for Biotechnology Information (NCBI), and at the species level, *C. verruculosa* KHW-7 is the first sequenced genome. The 31.59 Md genome of *C. verruculosa* KHW-7 contained a total of 9,877 genes, which is somehow lower compared to other *Curvularia* species, *C. lunata* W3 (33.5 Mb, 10,165 protein-coding genes), *C. kusanoi* 30 M1 (33.3 Mb, 11,004 protein-coding genes), and *Curvularia* sp. IFB-Z10 (33 Mb, 9,469 protein-coding genes) (Quach et al., 2022). The presence of repetitive components, such as interspersed repetitions and low-complexity DNA sequences, was detected in the genome assembly of *C. verruculosa* KHW-7. However, a high repetitive content might be associated with accelerated species evolution (Pisupati et al., 2018).

TEs are mobile genetic units that can induce mutations, alter gene expression, and cause chromosomal rearrangements (Castanera et al., 2016; Lorrain et al., 2021). These processes contribute to the successful adaptation of populations to environmental changes. *Phytophthora infestans* and *Blumeriagrami* f. sp. hordei are two major plant infections with large genome sizes due to the presence of a large number of TEs, which make up approximately 29% of the genome (Haas et al., 2009; Spanu et al., 2010). Furthermore, the TE repertoires exhibit variations not just at the genus level but also among closely related fungal taxa. In addition, TEs also serve as unique promoters that disrupt transcription processes, hence playing a significant role in fungal development and evolution (Mita and Boeke, 2016). The genetic analysis detected a total of 2048 transmembrane helices related to 30 significantly enriched transcription factors in *C. verruculosa* KHW-7 (Figure 9). Moreover, the KEGG analysis has identified 142 genes related to environmental information processing (Table 3). Therefore, this discovery has the potential to facilitate the examination of the evolutionary connections and lifestyle modifications of *C. verruculosa* KHW-7 across numerous ecological habitats that have yet to be studied.

In *C. verruculosa* KHW-7, 509 genes were found to be related to carbohydrate enzymes (CAZymes) (Figure 7). CAZymes play major roles in plant polysaccharide degradation (Ospina-Giraldo et al., 2010). Therefore, investigating and analyzing CAZymes from fungi with distinct methods of nourishment or infection mechanisms can yield insights into their lifestyles and infection patterns (Zhao et al., 2013).

## Conclusion

In this study, a high-quality *de novo* genome of the fungal isolate *C. verruculosa* KHW-7 was obtained via WGS and assembly. As per NCBI genome submission status, this is the first WGS sequencing of *C. verruculosa*. WGS has revolutionized fungal characterization by providing a holistic view of their genetic blueprint. From our findings, important genome features and annotations were produced using various open-source tools and databases. The discovery not only aids in understanding the biology and evolution of *C. verruculosa* but also holds immense potential for guiding disease management strategies.

## Data availability statement

The datasets presented in this study can be found in online repositories. The names of the repository/repositories and accession number(s) can be found at: https://www.ncbi.nlm.nih.gov/, PRJNA1023514.

## Author contributions

PB: Writing – review & editing, Writing – original draft, Methodology, Investigation, Formal analysis. SI: Writing – review & editing, Writing – original draft, Methodology, Investigation, Data curation. AM: Writing – review & editing, Resources, Methodology, Investigation, Formal analysis. HM: Writing – review & editing, Formal analysis, Software, Investigation. SA: Writing – review & editing, Validation, Methodology, Formal analysis. MA: Writing – review & editing, Validation, Data curation, Formal analysis. VY: Writing – review & editing, Validation, Software, Methodology, Data curation. AP: Writing – review & editing, Writing – original draft, Visualization, Supervision, Conceptualization. MJ: Writing – review & editing, Software, Methodology, Formal analysis, Data curation. DS: Writing – review & editing, Software, Resources, Formal analysis, Data curation. HB: Writing – review & editing, Writing – original draft, Visualization, Supervision, Project administration.

## Footnotes

^1^ https://github.com/EVidenceModeler

^2^ https://geneontology.org/

^3^ http://eggnog45.embl.de/#/app/home

^4^ https://apps.cytoscape.org/apps/cluego

^5^ https://phobius.sbc.su.se/

^6^ https://fungismash.secondarymetabolites.org/#!/start

^7^ http://lowelab.ucsc.edu/tRNAscan-SE/

^8^ https://bio.tools/barrnap

^9^ https://github.com/nextgenusfs/funannotate

^10^ https://www.ncbi.nlm.nih.gov/refseq/about/nonredundantproteins/

^11^ http://www.phi-base.org/

^12^ https://card.mcmaster.ca/

## Supplementary material

SUPPLEMENTARY FIGURE S1 Sample collection site. Blue spot indicates sample collection site from Gujarat province, India with location coordinates.

SUPPLEMENTARY FIGURE S2 Number of *Curvularia verruculosa* gene sequences with annotations from different databases.

SUPPLEMENTARY FIGURE S3 ClueGO network analysis results of the significantly enriched GO terms (molecular functions).

SUPPLEMENTARY FIGURE S4 ClueGO network analysis results of the significantly enriched GO terms (cellular components).

SUPPLEMENTARY FIGURE S5 Biosynthetic gene cluster identified by antiSMASH.
