# Supplementary figures and images for: Whole-genome sequencing of marine water-derived Curvularia verruculosa KHW-7: a pioneering study
Source: Front Microbiol. 2024 May 23;15:1363879. doi: 10.3389/fmicb.2024.1363879 (PMC11155457; doi:10.3389/fmicb.2024.1363879)

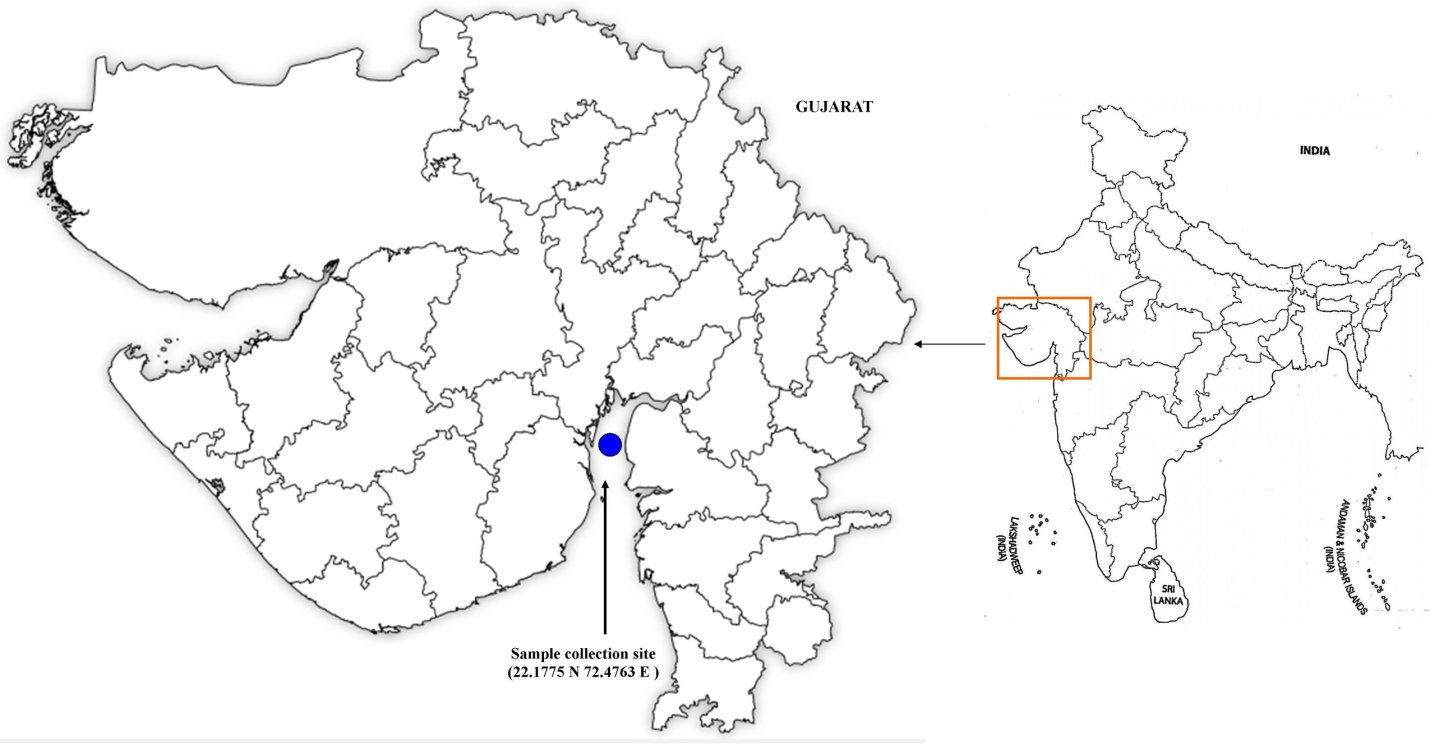

Supplement: SUPPLEMENTARY FIGURE S1 — Sample collection site. Blue spot indicates sample collection site from Gujarat province, India with location coordinates. [file Image_1.TIF]

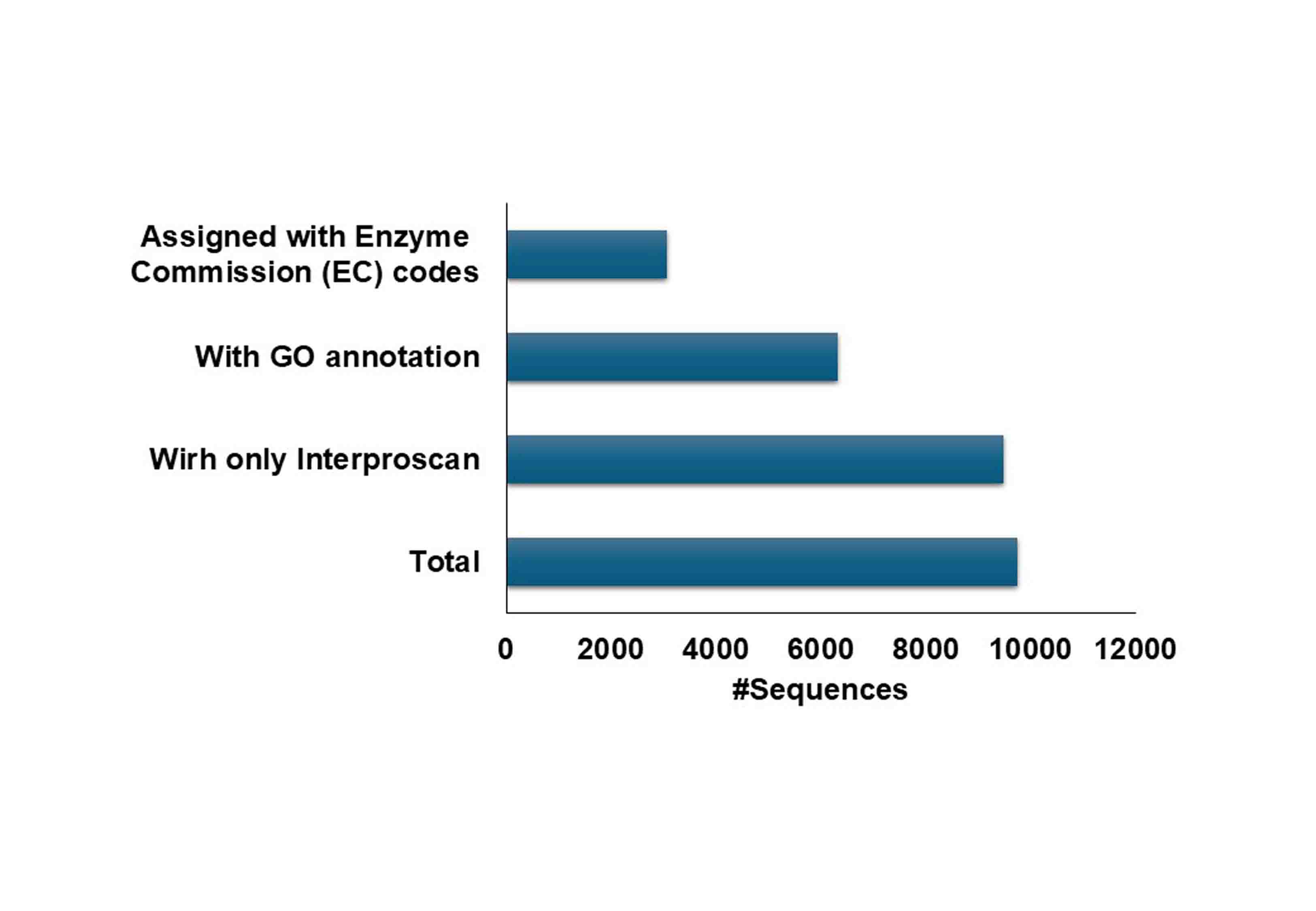

Supplement: SUPPLEMENTARY FIGURE S2 — Number of Curvularia verruculosa gene sequences with annotations from different databases. [file Image_2.JPEG]

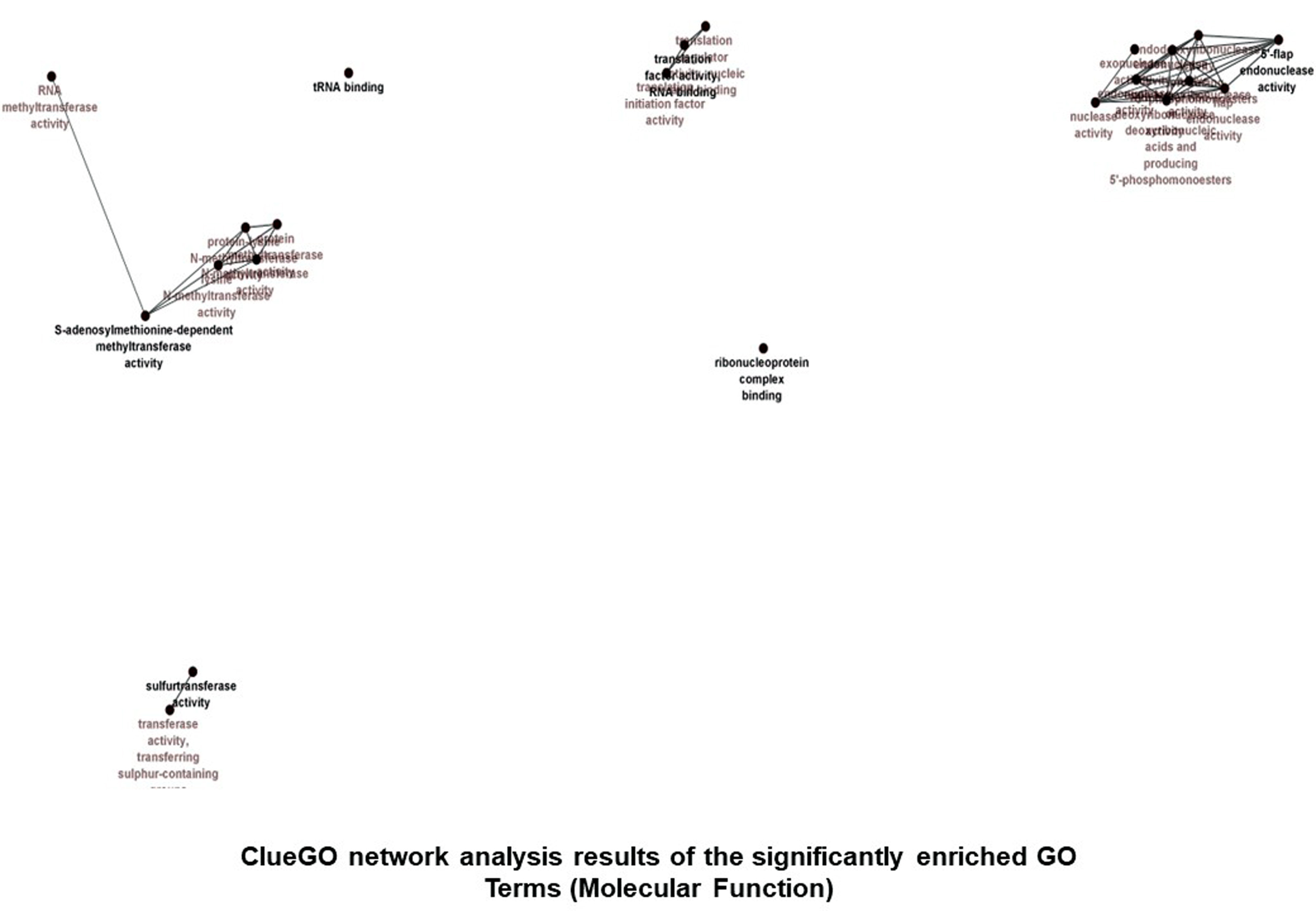

Supplement: SUPPLEMENTARY FIGURE S3 — ClueGO network analysis results of the significantly enriched GO terms (molecular functions). [file Image_3.JPEG]

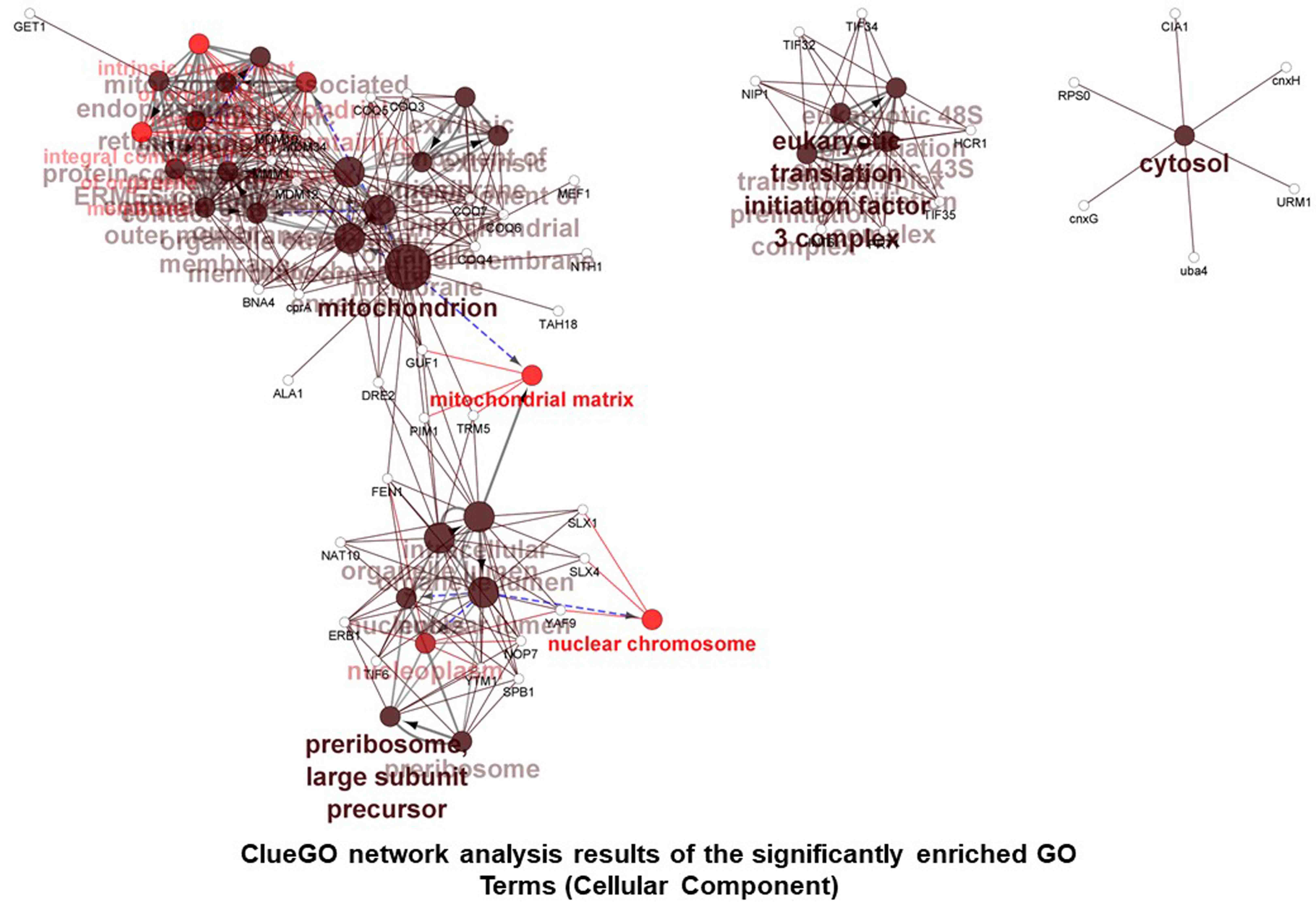

Supplement: SUPPLEMENTARY FIGURE S4 — ClueGO network analysis results of the significantly enriched GO terms (cellular components). [file Image_4.JPEG]

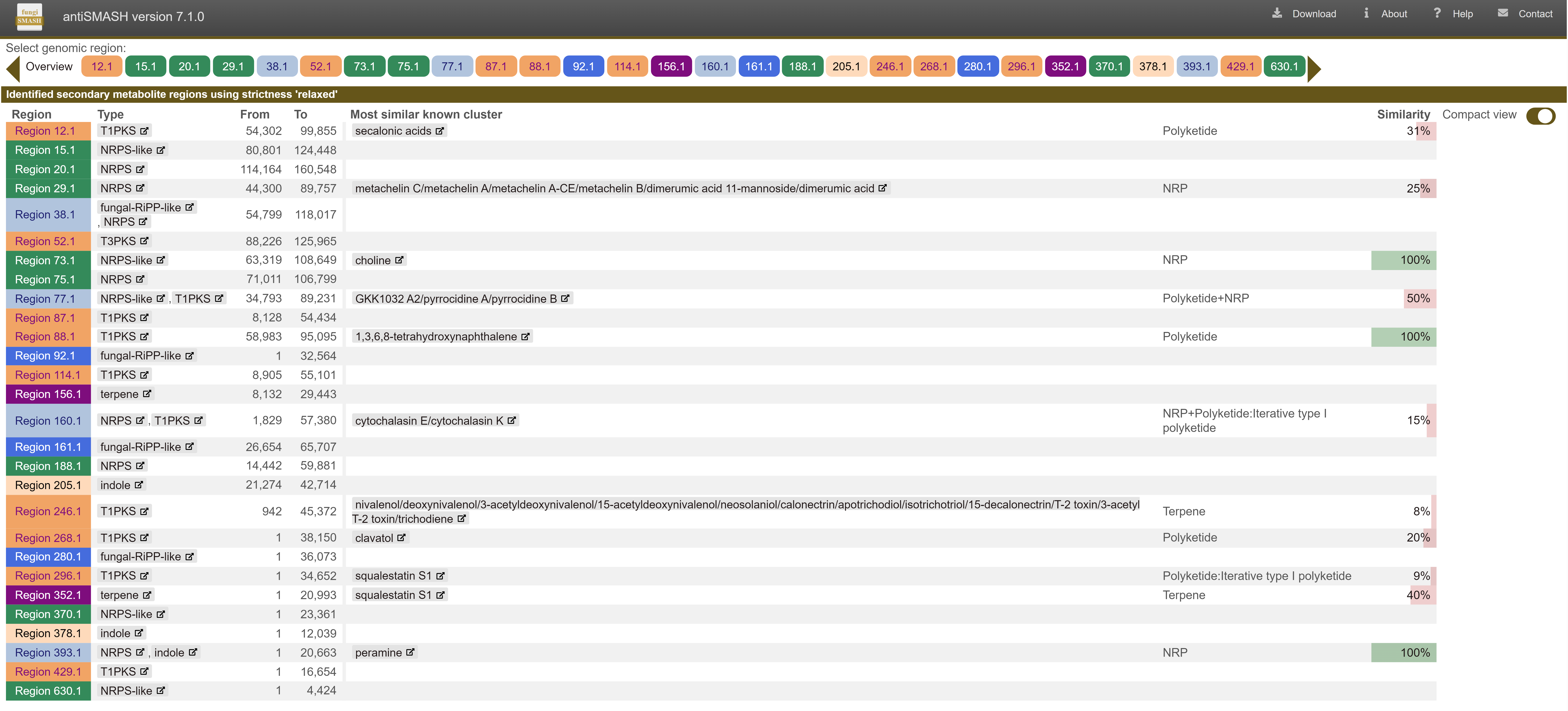

Supplement: SUPPLEMENTARY FIGURE S5 — Biosynthetic gene cluster identified by antiSMASH. [file Image_5.PNG]
