# Supplementary material for: Whole-genome sequencing of marine water-derived Curvularia verruculosa KHW-7: a pioneering study
Source: Front Microbiol. 2024 May 23;15:1363879. doi: 10.3389/fmicb.2024.1363879 (PMC11155457; doi:10.3389/fmicb.2024.1363879)
Supplement: Supplementary file 6 [file Table_1.docx]

**Supplementary Table S1: Comparison of genome features between** ***C. verruculosa* KHW-7and** **other *Curvularia* species**

| **Features** | ***C. verruculosa* KHW-7** | ***C. lunata*CX-3** | ***C. lunata*m118** |
| --- | --- | --- | --- |
| Assembly size (Mb) | 31.59 | 35.5 | 31.2 |
| Scaffolds | 1323 | 340 | 171 |
| GC (%) | 50.44 | 50.22 | 50.9 |
| Protein-coding genes | 9877 | 11,234 | 11,004 |
| Mean gene length (Bp) | 1638.3 | 1,448 | 1,429 |
| Secreted proteins | 9745 | 840 | 834 |
